# The effect of SGLT2i on in-hospital acute heart failure risk in acute myocardial infarction patients—a retrospective study

**DOI:** 10.3389/fcvm.2023.1158507

**Published:** 2023-05-16

**Authors:** Yi Zhu, Jia-li Zhang, Hong Jin, Yuan Ji, Fang-fang Wang

**Affiliations:** ^1^ Department of Cardiology, The Affiliated Changzhou No.2 People’s Hospital of Nanjing Medical University, Changzhou, China; ^2^ Department of Gastroenterology Centre, The Affiliated Changzhou No.2 People’s Hospital of Nanjing Medical University, Changzhou, China; ^3^ Department of Cardiology, Zhongda Hospital, School of Medicine, Southeast University, Nanjing, China

**Keywords:** SGLT2I, AHF, AMI, NSTEMI, hospitalization

## Abstract

**Background and aims:**

The roles of sodium-glucose cotransporter 2 inhibitor (SGLT2i) in acute heart failure (AHF) risk after acute myocardial infarction (AMI) remain unclear. In this study, we explored the correlation between SGLT2i administration and short-term in-hospital AHF risk in AMI patients.

**Methods:**

This single-center, retrospective, and observational study included 990 AMI patients comprising 386 non-ST-segment elevation myocardial infarction (NSTEMI) and 604 segment elevation myocardial infarction (STEMI) patients enrolled from January 2019 to March 2022. Demographic information, clinical characteristics, medical treatment, and laboratory examination results during hospitalization were extracted from an electronic medical record system. The primary outcome was defined as all-cause AHF during hospitalization.

**Results:**

In NSTEMI patients, a significantly lower proportion received SGLT2i treatment in the AHF group compared with the non-AHF group. During hospitalization, SGLT2i significantly reduced brain natriuretic peptide levels both in STEMI and NSTEMI patients. Multivariate logistic regression and stratification analyses suggested that SGLT2i is associated with reduced in-hospital AHF risk, and has a strong protective effect against AHF in NSTEMI patients with hypertension. Furthermore, SGLT2i significantly reduced the risk of in-hospital AHF for both patients with diabetes and non-diabetes.

**Conclusions:**

SGLT2i can reduce the risk of AHF in AMI patients during hospitalization.

## Introduction

Acute myocardial infarction (AMI) is a serious and fatal cardiovascular emergency. Rupture of vulnerable coronary plaques can result in thrombosis, leading to complete or partial coronary artery occlusion, and eventually causing myocardial ischemia or necrosis. With advances in coronary intervention technology and the standardization of admission process for patients with chest pain, the mortality rate of AMI patients has been considerably reduced, and the complications caused by necrotic myocardial tissue have been greatly decreased. However, the adverse cardiovascular events such as acute heart failure (AHF) and arrhythmia that occur among in-hospital AMI patients still pose a serious burden on postoperative management and the rational allocation of medical resources (1, 2).

Studies have confirmed that sodium-glucose cotransporter 2 inhibitor (SGLT2i) can significantly improve cardiovascular and renal outcomes (3). A meta-analysis of randomized trials reveals that SGLT2i reduce mortality and morbidity in patients with heart failure (4). Although current guidelines generally recommend that SGLT2i should be discontinued during AMI, a recent JACC report highlighted the potential for improved patient outcomes through early application of SGLT2i in AMI (5). The safety issue of SGLT2i for type 2 diabetes mellitus (T2DM) patients combined with AMI deserves our attention. SGLT2i treatment may result in an asymptomatic increase in blood ketone body levels, but the vast majority of patients can compensate for this slight increase in ketone body levels. From the perspective of mechanism, SGLT2i related diabetes ketoacidosis can be predicted, prevented and controlled. The increase in ketone body levels is a metabolic adaptation of the body to glucose loss. In the presence of metabolic stress, especially in patients with diabetes and heart failure, the ketone body energy supply is more efficient and plays a protective role in the heart (6). Due to differences in the number and extent of lesions and emergency treatment strategies between ST-segment elevation myocardial infarction (STEMI) and non-ST-segment elevation myocardial infarction (NSTEMI) patients, the mechanism and incidence of AHF during hospitalization are also different. At present, the discrepancies in the protection provided by SGLT2i against AHF in STEMI or NSTEMI patients during hospitalization are unclear.

In this retrospective study, we aimed to investigate the effect of SGLT2i intervention on HF indicators in hospitalized STEMI and NSTEMI patients, and to explore the correlation between SGLT2i administration and short-term risk of AHF during hospitalization in AMI patients.

## Patients and methods

### Participants

This study was performed in compliance with the Declaration of Helsinki, and was approved by the Committee of Clinical Investigation of The Affiliated Changzhou No.2 People's Hospital of Nanjing Medical University (KY314-01).

**Figure 1:**
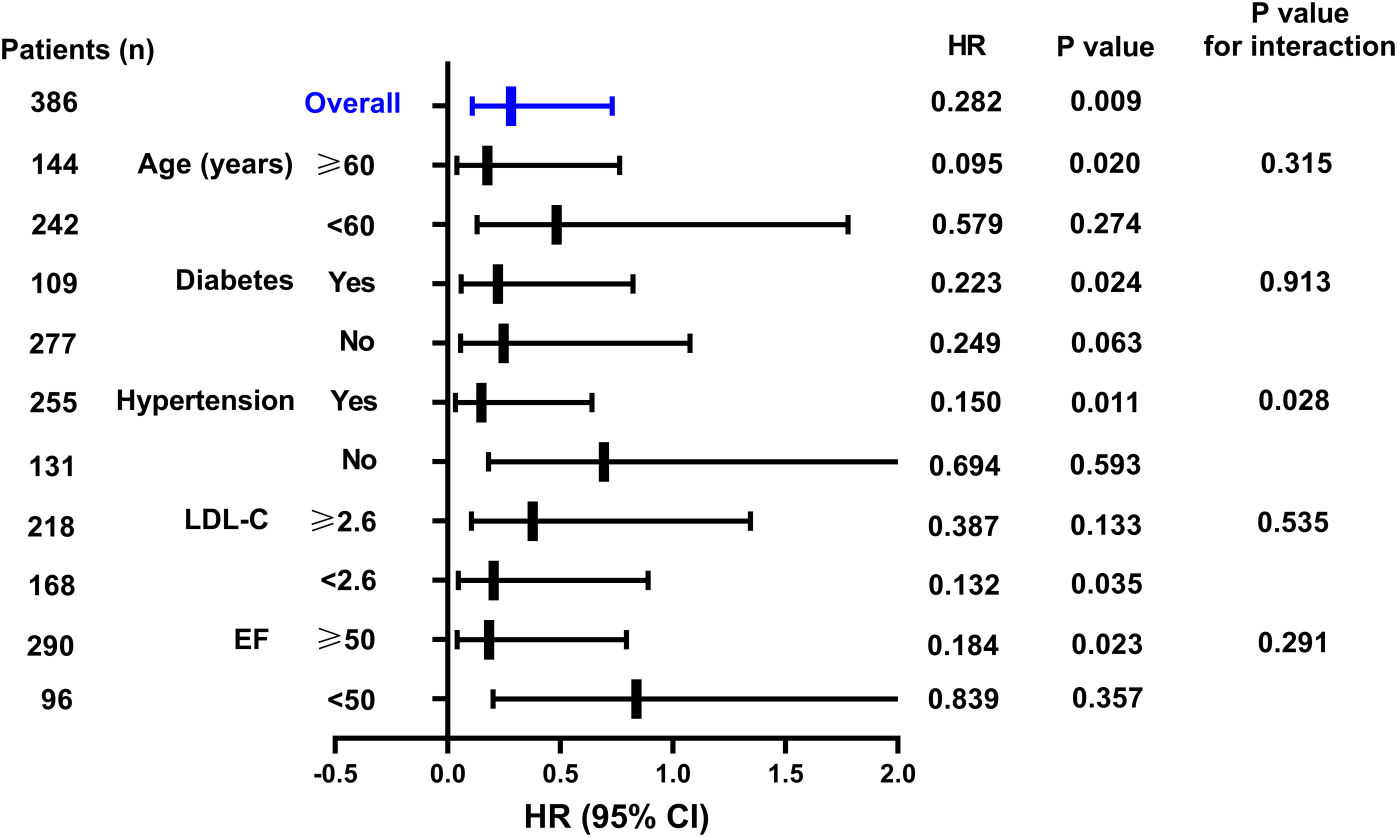
Hazard ratios of the SGLT2i for predicting in-hospital AHF in NSTEMI group in the subgroup analysis..

This single-center, retrospective, and observational study was registered in the China Clinical Trial Registration Center (ChiCTR2300067892). In total, 990 patients comprising 386 NSTEMI and 604 STEMI patients admitted to the Affiliated Changzhou No.2 People's Hospital of Nanjing Medical University from January 2019 to March 2022 were enrolled in this study. The research protocol was shown in Figure 1.

The inclusion criteria were as follows: (1) age range 18–80 years, (2) admitted diagnosis of AMI, including STEMI and NSTEMI. Diagnostic criteria of STEMI: (1) a history of chest pain/discomfort; (2) at admission, continuous elevation of ST segments in two or more adjacent ECG leads ≥0.1 mV (>30 min, V2, V3 ≥ 0.2 mV) or new onset of left bundle branch block; (3) myocardial injury markers (troponin, CK-MB) increased beyond the 99th percentile of the laboratory reference limit. Diagnostic criteria of NSTEMI: (1) a history of chest pain/discomfort; (2) in leads with R wave dominant or R/S > 1, new horizontal or downwardly inclined ST segment depression ≥0.05 mV or T wave inversion ≥0.1 mV appears in two adjacent leads; (3) myocardial injury markers (troponin, CK-MB) increased beyond the 99th percentile of the laboratory reference limit.

The patient exclusion criteria were as follows: (1) patients had previous history of heart failure, (2) patients received SGLT2i before, (3) malignant tumor, (4) pregnancy, (5) severe liver dysfunction, (6) severe hematological disorders, (7) history of coronary artery bypass grafting, (8) cardiogenic shock, (9) mechanical ventilation, and (10) mechanical circulatory support. Severe liver dysfunction was diagnosed as the elevated serum transaminases, severely elevated serum bilirubin, decreased albumin concentration, and coagulation disorders. Severe hematological disorders were considered as multiple myeloma, lymphoma, myelodysplastic syndrome, or leukemia.

### Data collection and definition

Information relating to demographics, clinical characteristics, clinical events, medical histories, medical treatment, laboratory examinations, and imaging records collected during hospitalization were extracted from an electronic medical record system.

The primary end-point was in-hospital AHF, and the second end-points were BNP levels and in-hospital arrhythmia. AHF was diagnosed based on typical symptoms, signs, and laboratory tests of factors, such as orthopnea, acute pulmonary edema, and BNP levels. Considering that during the acute phase, AHF events and subsequent respiratory distress may result in discontinuation of feeding and avoidance of SGLT2i, only AHF events occurring 48 h after admission were recorded. Arrhythmia was defined as at least one episode of atrial fibrillation, atrial flutter, ventricular fibrillation, or ventricular flutter. Only arrhythmia events occurring 48 h after admission were recorded. The Gensini score was used to assess the severity of coronary artery disease and was calculated according to a previously described protocol (7).

### Statistical analysis

All data were tested for normal distribution. Approximately normally distributed data were expressed as the mean ± standard deviation and skewed continuous variables were expressed as the median (interquartile range). Continuous variables between two groups were compared using Student's *t*-test or the Mann–Whitney *U*-test. The *χ*^2^ test was used for comparisons of categorical variables between groups. Univariate and multivariate logistic regression analyses were performed to identify the predictive value of SGLT2i intervention for AHF or arrhythmia risk during hospitalization. All tests were two-sided. *P*-values <0.05 were considered statistically significant. Statistical analyses were performed using SPSS software 22.0 (SPSS Inc., Chicago, IL, USA).

## Results

Table 1 shows the clinical and biochemical characteristics of the 990 patients enrolled in this retrospective study. All-cause AHF was recorded for 38 of 604 STEMI and 51of 386 NSTEMI patients during hospitalization. Among the STEMI patients, the mean age of patients with AHF was significantly higher than that of patients without AHF. In addition, the proportion of concurrent arrhythmia in the AHF group was significantly higher than that in the non-AHF group for both STEMI and NSTEMI patients. The Gensini score was used to reflect the severity of coronary lesions. In NSTEMI patients, the Gensini score in the AHF group was significantly higher than that in the non-AHF group, while there was no significant difference among the STEMI patients. Comparison of biochemical test data revealed that in NSTEMI patients, the AHF group had lower HDL-C levels compared with the non-AHF group, and higher levels of myocardial injury and heart failure markers (creatine phosphokinase, creatine kinase-MB, hydroxybutyrate dehydrogenase, and BNP), while there were no significant differences among the STEMI patients. Furthermore, patients with NSTEMI and AHF had lower EF values than those without AHF. Finally, we analyzed the patients' drug interventions during hospitalization. Among NSTEMI patients, the proportions of patients receiving angiotensin receptor-neprilysin inhibitor (ARNI) and SGLT2i therapies in the AHF group were significantly lower than those in the non-AHF group, while the opposite trend was observed for mineralocorticoid receptor antagonist (MRA) treatment.

**Table 1:** Basic characteristics of enrolled AMI patients.

Characteristics	STEMI		*P* value	NSTEMI		*P* value
	Without AHF (*n* = 566)	With AHF (*n* = 38)		Without AHF (*n* = 335)	With AHF (*n* = 51)	
Age (years)	60.4 ± 14.2	64.9 ± 12.3	0.038	63.3 ± 13.1	65.6 ± 14.7	0.296
Sex, male, *n* (%)	459 (81.1)	31 (81.6)	0.941	241 (71.9)	33 (64.7)	0.289
BMI (kg/m^2^)	25.0 ± 4.1	25.1 ± 3.0	0.788	24.5 ± 4.2	24.2 ± 3.5	0.628
Smoking, *n* (%)	275 (50.9)	22 (59.5)	0.315	146 (46.8)	19 (38.8)	0.295
Hypertension, *n* (%)	342 (60.4)	28 (73.7)	0.104	218 (65.1)	37 (72.5)	0.294
Diabetes, *n* (%)	133 (23.5)	14 (36.8)	0.064	91 (27.2)	18 (35.3)	0.230
In-hospital arrhythmia, *n* (%)	28 (4.9)	17 (44.7)	<0.001	6 (1.8)	24 (47.1)	<0.001
Gensini score	49 (35–81)	53 (37–81)	0.690	29 (9–48)	60 (46–84)	< 0.001
**Biochemical test**						
ALP (U/L)	77.6 ± 21.3	76.3 ± 21.4	0.723	77.5 ± 26.0	80.4 ± 41.1	0.629
UA (umol/L)	328 (272–392)	344 (287–418)	0.417	335 (277–410)	353 (309–418)	0.135
LDL-C (mmol/L)	2.74 ± 0.84	2.82 ± 1.00	0.671	2.54 ± 0.92	2.79 ± 1.37	0.209
HDL-C (mmol/L)	1.03 ± 0.28	1.09 ± 0.33	0.330	1.05 ± 0.29	0.97 ± 0.22	0.020
TC (mmol/L)	4.5 ± 1.2	4.6 ± 1.1	0.327	4.3 ± 1.2	4.5 ± 1.5	0.328
TG (mmol/L)	1.47 (1.04–2.10)	1.75 (1.12–2.14)	0.518	1.52 (1.13–2.16)	1.45 (1.17–1.90)	0.779
CPK (U/L)	950 (312–2,003)	1,132 (528–1,791)	0.517	154 (76–376)	236 (116–879)	0.010
CK-MB (U/L)	81 (35–167)	102 (47–144)	0.598	22 (16–42)	31 (19–78)	0.008
HBDH (U/L)	483 (276–824)	493 (349–718)	0.662	197 (151–324)	274 (188–444)	0.001
BNP (pg/mL)	291 (81–1,165)	394 (79–1,445)	0.703	456 (149–1,610)	913 (284–3,193)	0.006
HbA1c (%)	6.5 ± 1.5	6.9 ± 1.7	0.198	6.5 ± 1.5	6.8 ± 1.7	0.262
Ccr (ml/min)	69 ± 43	71 ± 33	0.775	63 ± 31	59 ± 29	0.466
**Ultrasonic cardiogram**						
LA (mm)	3.9 ± 0.5	3.9 ± 0.4	0.825	3.9 ± 0.5	4.0 ± 0.5	0.198
Diastole LV (mm)	5.3 ± 2.2	5.2 ± 0.6	0.571	5.2 ± 0.6	5.3 ± 0.6	0.142
EF (%)	50 ± 9	52 ± 8	0.159	55 ± 9	50 ± 13	0.020
**Pharmacological intervention**						
Double antiplatelet, *n* (%)	521 (92.4)	37 (97.4)	0.694	291 (87.4)	48 (94.1)	0.432
Anticoagulation, *n* (%)	19 (3.4)	0 (0)	0.493	11 (3.3)	2 (3.9)	0.829
β-block, *n* (%)	340 (60.1)	21 (55.3)	0.550	176 (52.5)	29 (56.9)	0.578
Statin, *n* (%)	559 (98.8)	37 (97.4)	0.382	331 (98.8)	51 (100)	0.497
ACEI/ARB, *n* (%)	176 (31.1)	12 (31.6)	0.956	106 (31.6)	14 (27.5)	0.538
ARNI, *n* (%)	150 (26.5)	10 (26.3)	0.944	107 (32.3)	8 (16.0)	0.019
SGLT2i, *n* (%)	131 (23.2)	5 (13.1)	0.151	93 (27.8)	5 (9.8)	0.006
MRA, *n* (%)	114 (20.1)	9 (23.7)	0.611	50 (14.9)	16 (31.4)	0.004

Values are shown as the means ± SD, median (interquartile range) or percentage.

BMI, body mass index; ALP, alkaline phosphatase; UA, uric acid; LDL-C, low-density lipoprotein cholesterol; HDL-C, high-density lipoprotein cholesterol; TC, total cholesterol; TG, triglycerides; CPK, creatine phosphokinase; CK-MB, creatine kinase-MB; HBDH, hydroxybutyrate dehydrogenase; BNP, brain natriuretic peptide; Ccr, creatinine clearance rate; LA, left atrium; LV, left ventricle; EF, ejection factor; ARNI, angiotensin receptor-neprilysin inhibitor; SGLT2i, sodium-glucose cotransporter 2 inhibitor; MRA, mineralocorticoid receptor antagonist.

Arrhythmia: at least one of atrial fibrillation, atrial flutter, ventricular fibrillation, and ventricular flutter.

Since BNP tests are performed to evaluate cardiac function and prognosis for in-hospital AMI patients, we grouped STEMI and NSTEMI patients according to whether SGLT2i was used. In total, 462 patients (BNP >400 ng/ml, day 1) were enrolled, comprising 258 patients in the STEMI group (59 in the SGLT2i group and 199 in the non-SGLT2i group) and 204 patients in the NSTEMI group (40 in the SGLT2i group and 164 in the non-SGLT2i group). Table 2 showed that SGLT2i administration markedly decreased the proportion of patients whose BNP levels were over 400 ng/ml, compared with effects observed in the SGLT2i-free groups.

**Table 2:** SGLT2i significantly reduced the proportion of patients with BNP > 400 ng/ml.

	STEMI patients		*P* value	NSTEMI patients		*P* value
	Without SGLT2i	With SGLT2i		Without SGLT2i	With SGLT2i	
BNP > 400 pg/ml (day 1), *n* (%)	199 (100)	59 (100)		164 (100)	40 (100)	
BNP > 400 pg/ml (after intervention), *n* (%)	75 (37.7)	9 (15.3)	0.002	63 (38.4)	6 (15.0)	0.008

Multivariate logistic regression analyses showed that SGLT2i therapy was associated with reduced in-hospital AHF risk in STEMI patients (*P* < 0.05 for models 3–5) (Table 3). In both univariate and multivariate logistic regression analyses, SGLT2i intervention was associated with a reduction in the risk of AHF occurrence during hospitalization of NSTEMI patients (*P* < 0.05 for models 1–4) (Table 3). In both univariate and multivariate logistic regression analyses, SGLT2i was not associated with reduced in-hospital arhythmia risk in STEMI and NSTEMI patients (*P* > 0.05 for model 1–5) (Table 4).

**Table 3:** Univariate and multivariate logistic analyses for the in-hospital AHF risk according to the SGLT2i administration.

STEMI		OR	95% CI	*P* value	NSTEMI		OR	95% CI	*P* value
Model 1	SGLT2i-free	Reference			Model 1	SGLT2i-free	Reference		
	SGLT2i	0.501	0.192–1.309	0.158		SGLT2i	0.282	0.109–0.731	0.009
Model 2	SGLT2i-free	Reference			Model 2	SGLT2i-free	Reference		
	SGLT2i	0.545	0.207–1.434	0.219		SGLT2i	0.271	0.104–0.708	0.008
Model 3	SGLT2i-free	Reference			Model 3	SGLT2i-free	Reference		
	SGLT2i	0.263	0.088–0.784	0.017		SGLT2i	0.314	0.116–0.849	0.022
Model 4	SGLT2i-free	Reference			Model 4	SGLT2i-free	Reference		
	SGLT2i	0.237	0.078–0.723	0.011		SGLT2i	0.294	0.101–0.853	0.024
Model 5	SGLT2i-free	Reference			Model 5	SGLT2i-free	Reference		
	SGLT2i	0.219	0.069–0.691	0.010		SGLT2i	0.338	0.114–0.999	0.050

Model 1: Unadjusted.

Model 2: Adjusted for age, sex.

Model 3: Model 2 + smoking, hypertension, diabetes, cerebral infarction.

Model 4: Model 3 + Ccr, HDL-C, LDL-C, TC, TG, CPK, CK-MB, HBDH, BNP, HbA1c.

Model 5: Model 4 + β-block at discharge, ACEI/ARB at discharge, ARNI at discharge, MRA at discharge.

**Table 4:** Univariate and multivariate logistic analyses for the in-hospital arrhythmia risk according to the SGLT2i administration.

STEMI		OR	95% CI	*P* value	NSTEMI		OR	95% CI	*P* value
Model 1	SGLT2i-free	Reference			Model 1	SGLT2i-free	Reference		
	SGLT2i	0.846	0.397–1.804	0.666		SGLT2i	0.715	0.283–1.803	0.477
Model 2	SGLT2i-free	Reference			Model 2	SGLT2i-free	Reference		
	SGLT2i	0.898	0.416–1.939	0.784		SGLT2i	0.706	0.278–1.795	0.465
Model 3	SGLT2i-free	Reference			Model 3	SGLT2i-free	Reference		
	SGLT2i	0.508	0.209–1.234	0.135		SGLT2i	0.804	0.302–2.144	0.664
Model 4	SGLT2i-free	Reference			Model 4	SGLT2i-free	Reference		
	SGLT2i	0.455	0.174–1.189	0.108		SGLT2i	0.922	0.309–2.750	0.885
Model 5	SGLT2i-free	Reference			Model 5	SGLT2i-free	Reference		
	SGLT2i	0.449	0.165–1.218	0.116		SGLT2i	1.034	0.341–3.135	0.954

Model 1: Unadjusted.

Model 2: Adjusted for age, sex.

Model 3: Model 2 + smoking, hypertension, diabetes, cerebral infarction.

Model 4: Model 3 + Ccr, HDL-C, LDL-C, TC, TG, CPK, CK-MB, HBDH, BNP, HbA1c.

Model 5: Model 4 + β-block at discharge, ACEI/ARB at discharge, ARNI at discharge, MRA at discharge.

**Figure 2:**
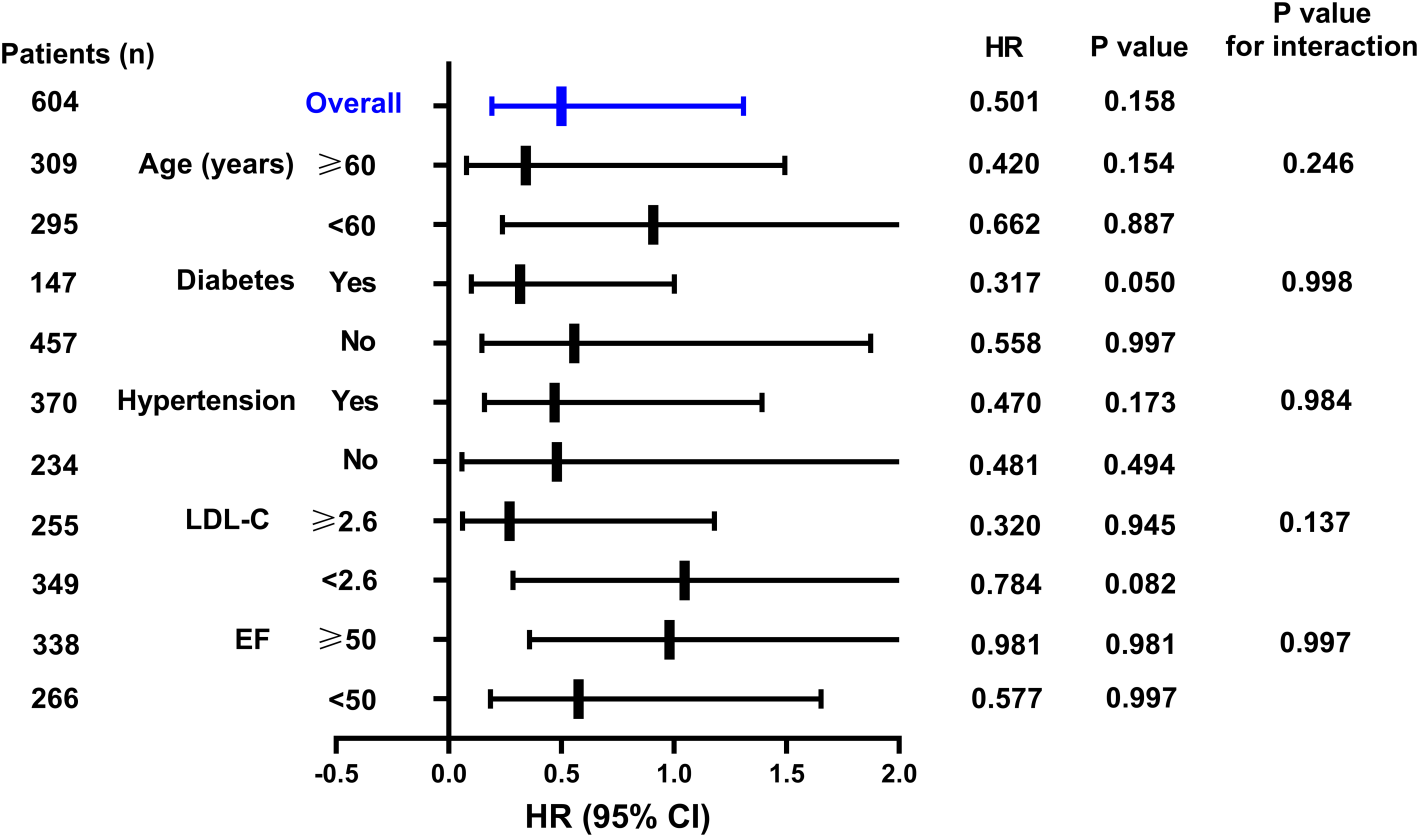
Hazard ratios of the SGLT2i for predicting in-hospital AHF in NSTEMI group in the subgroup analysis.

As shown in Figure 2, when stratified by age, for patients aged ≥60 years, the in-hospital AHF occurrence rate in the SGLT2i group was increased by 0.177-fold (95% CI: 0.041–0.764, *P* = 0.020) compared to that in the SGLT2i-free group. For diabetic patients, the in-hospital AHF risk in the SGLT2i group was 0.223-fold lower than that in the SGLT2i-free group (95% CI: 0.060–0.824, *P* = 0.024), although the prediction values in non-diabetic patients were not statistically significant. For patients with hypertension, the in-hospital AHF risk in the SGLT2i group was 0.150-fold lower than that in the SGLT2i-free group (95% CI: 0.035–0.641, *P* = 0.011). The prediction values in patients without hypertension were not statistically significant, and the *P* value for interaction was 0.028. For patients with LDL-C < 2.6 mmol/L, the in-hospital AHF risk in the SGLT2i group was 0.204-fold lower than that in the SGLT2i-free group (95% CI: 0.047–0.891, *P* = 0.035), while the prediction values in patients (LDL-C ≥ 2.6 mmol/L) were not statistically significant. For patients with EF ≥50%, the in-hospital AHF risk in the SGLT2i group was 0.184-fold lower than that in the SGLT2i-free group (95% CI: 0.043–0.794, *P* = 0.023), However, in EF <50% subgroup, there was no significant difference between the SGLT2i and SGLT2i-free groups in terms of in-hospital AHF risk prediction.

**Figure 3:**
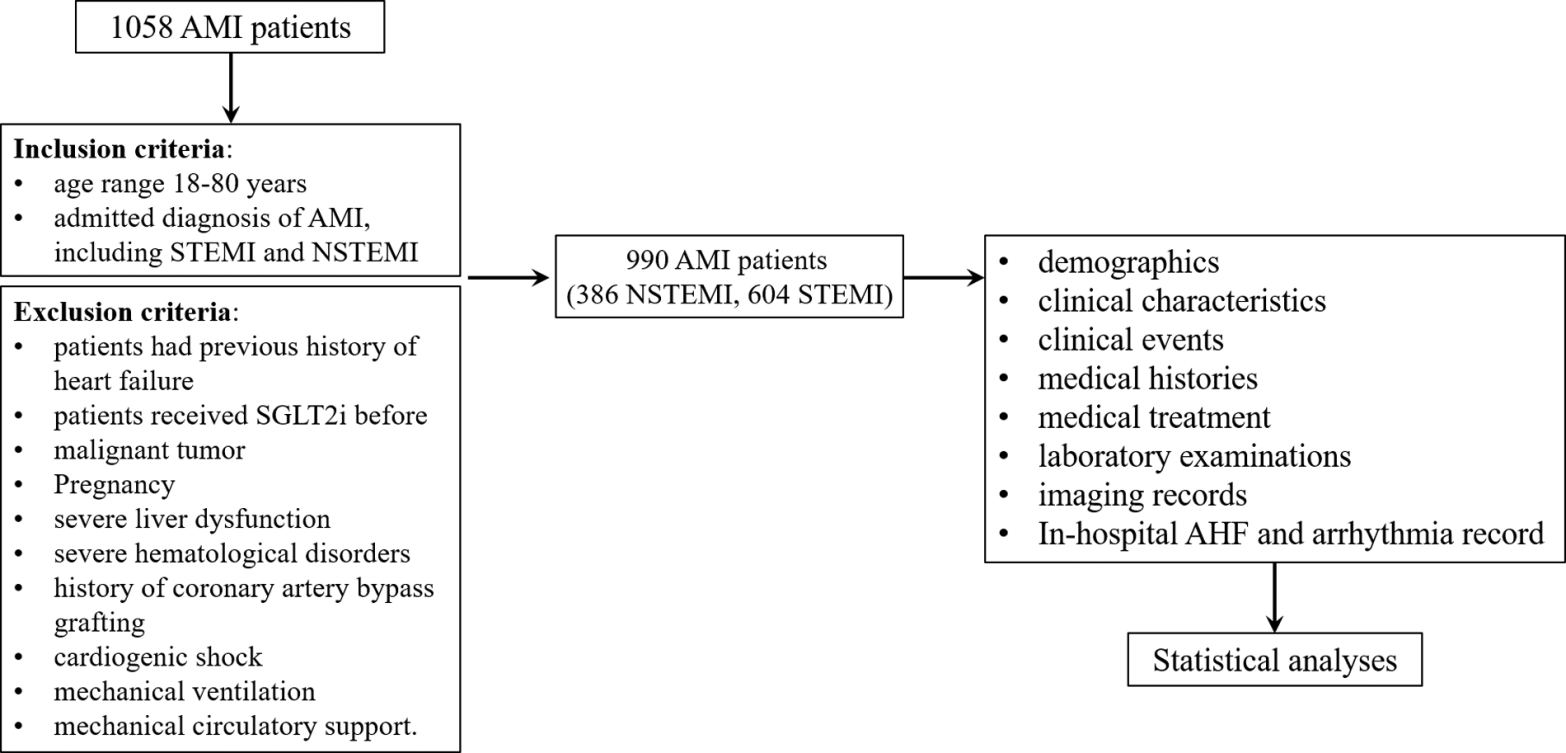
Study flow.

As shown in Figure 3, after stratifying STEMI patients by age (<60 or ≥60 years), diabetes (yes or no), hypertension (yes or no), LDL-C (<2.6 or ≥2.6) and EF (<50% or ≥50%), the AHF risk prediction values for SGLT2i treatment remained statistically insignificant.

Furthermore, we explored the effect of SGLT2i on the occurrence of in-hospital AHF and in-hospital arrhythmia in diabetics or non-diabetics. Table 5 demonstrated that SGLT2i could reduce the occurrence of in-hospital AHF both in diabetics and non-diabetics. However, for the occurrence of in-hospital arrhythmia, there was no significant difference between diabetics and non-diabetics.

**Table 5:** SGLT2i significantly reduced the risk of in-hospital AHF for both diabetics and non-diabetics.

	Diabetics		*P* value	Non-diabetics		*P* value
	Without SGLT2i	With SGLT2i		Without SGLT2i	With SGLT2i	
In-hospital AHF, *n* (%)	24 (20)	8 (5.9)	0.001	55 (8.7)	2 (2.0)	0.010
In-hospital arrhythmia, *n* (%)	18 (15)	12 (8.9)	0.094	42 (6.6)	3 (3.0)	0.117

Considering that part of the included patients used ARNI, which may have a synergistic effect with SGLT2i, we finally analyzed the impact of ARNI use on the SGLT2i effect. Table 6 suggested that ARNI use has no significant effect on SGLT2i reducing the risk of AHF in both STEMI and NSTEMI patients.

**Table 6:** ARNI had no significant effect on SGLT2i reducing the risk of AHF in both STEMI and NSTEMI patients.

STEMI	OR	95% CI	*P* value	NSTEMI	OR	95% CI	*P* value
SGLT2i (absence of ARNI)	Reference			SGLT2i (absence of ARNI)	Reference		
SGLT2i (presence of ARNI)	0.510	0.081–3.201	0.472	SGLT2i (presence of ARNI)	0.382	0.062–2.370	0.301

## Discussion

In this retrospective study of 990 patients, we found in NSTEMI patients, the proportion of SGLT2i intervention in the AHF group was significantly lower than that in the non-AHF group. In addition, we found that SGLT2i significantly reduced the BNP levels of STEMI and NSTEMI patients. SGLT2i was also found to be associated to improve the outcome for in-hospital AHF risk both in STEMI and NSTEMI patients, and had strong protective effects against in-hospital AHF risk in NSTEMI patients with hypertension. Furthermore, SGLT2i significantly reduced the risk of in-hospital AHF for both diabetics and non-diabetics.

Patients with AMI are at high risk of HF, severe arrhythmia, and cardiovascular death. Despite significant advances in early treatment strategies for myocardial infarction, patients are still exposed to residual cardiovascular risks associated with current drug treatments, especially in the critical early stage after AMI, when it is necessary to prevent adverse cardiac remodeling, AHF, and cardiovascular death (8). According to domestic and foreign guidelines and consensuses, SGLT2i can be used for patients with chronic HF with and without diabetes as well as those with chronic kidney disease (9, 10). SGLT2i can also be used in adult patients with reduced ejection fraction of heart failure (HFrEF) (NYHA II–IV) to decrease the risk of cardiovascular death and hospitalization for HF, regardless of whether the patient has diabetes (11). However, very few studies have been conducted on the effect of SGLT2i on the risk of in-hospital AHF in AMI patients.

In the initial analyses of basic clinical characteristics, we unexpectedly found that, among STEMI patients, there were no significant differences in basic clinical characteristics, biochemical analysis, echocardiographic results, and drug intervention between the AHF and non-AHF groups, with the exception of age and arrhythmia records. However, in NSTEMI patients, the AHF group had higher coronary severity, more significant traditional cardiovascular risk factors and indicators, and lower usage rate of ARNI and SGLT2i compared to the non-AHF group. It can be speculated that this difference of caused by most STEMI patients having relatively few coronary lesions, and therefore, emergency PCI is largely effective in preventing myocardial necrosis and subsequent complications, while in NSTEMI patients, coronary lesions are more diffuse, although the lesions are caused by non-transmural necrosis. Patients admitted to hospital are usually treated conservatively and with optional coronary interventions for some lesions. Therefore, patients with more significant traditional cardiovascular factors are prone to AHF. Thus, these results also suggested that the absence of ARNI or SGLT2i is, to some extent, related to the occurrence of AHF. Therefore, we then analyzed the effect of SGLT2i on BNP levels, and the results were as expected in that SGLT2i reduced BNP levels both in STEMI and non-STEMI patients. Although the previous study has shown that the protective effect of SGLT2i on HF does not depend on ARNI (12), our results suggested that ARNI has no significant effect on SGLT2i reducing the risk of AHF in both STEMI and NSTEMI patients, further indicating the protective effect of SGLT2i on AHF.

Logistic regression analyses suggested that SGLT2i treatment independently predicts the occurrence of AHF both in STEMI and NSTEMI patients. Logistic regression analyses for in-hospital AHF prediction showed that the HR of the SGLT2i group was lower than that of the SGLT2i-free group. Since AHF and arrhythmia were not consistent, the absence of statistical significance in the arrhythmia analysis was considered to be related to the small sample size. Furthermore, the stratified analysis suggested that, in NSTEMI patients, SGLT2i is more likely to reduce the risk of AHF in patients with hypertension, which reflected the specific cardiovascular protective mechanisms of SGLT2i. SGLT2i may not directly inhibit coronary thrombosis, but instead may inhibit neurohumoral activation (13), myocardial cell necrosis (14), and reperfusion injury (15). It may also enhance endothelial cell function and vasodilation (16), promote myocardial energy metabolism, maintain myocardial contractility (17), inhibit oxidative stress (18), improve coronary blood flow, and reduce ventricular load (19). These mechanisms of action may further prevent myocardial hypertrophy, myocardial fibrosis, and heart failure (20). SGLT2i may also have additional cardiometabolic benefits in high-risk groups following myocardial infarction, including reduced ventricular afterload and preload (21), improved glycemic control (22), and weight loss (23). Specifically, SGLT2i benefits the heart through mechanisms such as diuresis, natriuresis, reduction of inflammation and oxidative stress, promotion of red blood cell generation, inhibition of sympathetic nervous activity, improvement of cardiac energy metabolism and cardiac remodeling, and ultimately improvement of vascular function (24, 25). Therefore, in the face of extremely complex cardiac and vascular homeostasis disorders, SGLT2i can exert its unique cardiovascular protective effect to prevent the occurrence of AHF. Finally, we investigated the effect of SGLT2i on in-hospital AHF and the arrhythmia in diabetics or non-diabetics, which was consistent with previous reports, SGLT2i has an inhibitory effect on the occurrence of AHF in the non-diabetic patients. However, SGLT2i has no significant improvement effect on the occurrence of arrhythmia. From the data analysis, it can be seen that the use of SGLT2i can reduce the risk of the occurrence of arrhythmia. Further expanding the sample size may have different results.

There were some limitations in this study. As a single-center and retrospective study, bias caused by confounding factors was a prominent problem, although methods such as stratified analysis and both univariate and multivariate logistic regression analysis were used to eliminate the interference of confounding factors.

## Conclusions

In conclusion, SGLT2i can reduce the risk of AHF in AMI patients during hospitalization and is associated with a strong protective effect against AHF in NSTEMI patients with hypertension. SGLT2i significantly reduced the risk of in-hospital AHF for both diabetics and non-diabetics, which further suggests a protective role of SGLT2i in AMI.

## Data Availability

The raw data supporting the conclusions of this article will be made available by the authors, without undue reservation.
